# Chemosensory Loss: Functional Consequences of the World Trade Center Disaster

**DOI:** 10.1289/ehp.1001924

**Published:** 2010-05-18

**Authors:** Pamela H. Dalton, Richard E. Opiekun, Michele Gould, Ryan McDermott, Tamika Wilson, Christopher Maute, Mehmet H. Ozdener, Kai Zhao, Edward Emmett, Peter S.J. Lees, Robin Herbert, Jacqueline Moline

**Affiliations:** 1 Monell Chemical Senses Center, Philadelphia, Pennsylvania, USA; 2 New Jersey Department of Health and Senior Services, Trenton, New Jersey, USA; 3 University of Pennsylvania School of Medicine, Philadelphia, Pennsylvania, USA; 4 Johns Hopkins Bloomberg School of Public Health, Baltimore, Maryland, USA; 5 Mount Sinai School of Medicine, New York, New York, USA

**Keywords:** chemosensation, inflammation, irritation, occupational exposure, olfaction, World Trade Center

## Abstract

**Background:**

Individuals involved in rescue, recovery, demolition, and cleanup at the World Trade Center (WTC) site were exposed to a complex mixture of airborne smoke, dust, combustion gases, acid mists, and metal fumes. Such exposures have the potential to impair nasal chemosensory (olfactory and trigeminal) function.

**Objective:**

The goal of this study was to evaluate the prevalence of chemosensory dysfunction and nasal inflammation among these individuals.

**Methods:**

We studied 102 individuals who worked or volunteered at the WTC site in the days and weeks during and after 11 September 2001 (9/11) and a comparison group with no WTC exposure matched to each participant on age, sex, and job title. Participants were comprehensively evaluated for chemosensory function and nasal inflammation in a single session. Individual exposure history was obtained from self-reported questionnaires.

**Results:**

The prevalence of olfactory and trigeminal nerve sensitivity loss was significantly greater in the WTC-exposed group relative to the comparison group [prevalence ratios (95% confidence intervals) = 1.96 (1.2–3.3) and 3.28 (2.7–3.9) for odor and irritation thresholds, respectively]. Among the WTC responders, however, individuals caught in the dust cloud from the collapse on 9/11 exhibited the most profound trigeminal loss. Analysis of the nasal lavage samples supported the clinical findings of chronic nasal inflammation among the WTC-exposed cohort.

**Conclusions:**

The prevalence of significant chemosensory impairment in the WTC-exposed group more than 2 years after their exposure raises concerns for these individuals when the ability to detect airborne odors or irritants is a critical safety factor.

**Relevance to clinical practice:**

This outcome highlights the need for chemosensory evaluations among individuals with exposure to acute high or chronic levels of airborne pollutants.

Individuals who were near the World Trade Center (WTC) on 11 September 2001 (9/11) and those involved in rescue, demolition, and cleanup at the WTC site were exposed to a complex mixture of airborne smoke, dust, combustion gases, acid mists, and metal fumes. In the earliest days after the disaster, many may have worked without adequate respiratory protection. Since then, thousands of individuals have developed chronic respiratory symptoms related to their WTC exposure (Herbert et al. 2006; Landrigan et al. 2004).

As the sentinel portal to the respiratory system, the nose and upper respiratory tract subserve a number of critical functions in humans: warming and humidifying incoming air, trapping and desorbing particulates and vapors, and the sensory functions of olfaction (smell) and chemical somatosensation (irritation or chemesthesis) (Dalton and Opiekun 2006). These functions include a critical protective role, as the nasal epithelium has an unmatched ability to detect, trap, and detoxify many pollutants before their passage to the lower airways. This protective function does not come without cost, because exposure to many airborne pollutants and particles can induce upper airway inflammation and alter the sensory functions of the nose. Notably, however, evaluations of the impact of toxic exposures on the nose and nasal function are rarely performed in most occupational medicine clinics.

The potential for acute or chronic exposure to chemical vapors or particles to impair olfactory function has long been recognized [for a recent review, see Dalton (2010)]. Decrements in olfactory function have been found among occupationally exposed workers (Ählstrom et al. 1986; Schwartz et al. 1989, 1991) as well as community dwellers in urban areas with high levels of air pollution (Calderon-Garciduenas et al. 2010; Hudson et al. 2006). Such impairment portends serious consequences for the detection of many olfactory warning signals (e.g., smoke, spoiled food, and gas leaks) and a significant impact on nutritional status, eating satisfaction, and many other issues related to quality of life. In contrast, exposure-induced changes in upper airway somatosensation, mediated through activation of free nerve endings of the trigeminal nerve in nasal mucosa, have been less well documented. Chronic occupational exposure to irritants such as acetic acid or acetone specifically decreases trigeminal sensitivity to those chemicals (Dalton et al. 2006;Wysocki et al. 1997), whereas conditions such as allergic rhinitis, leading to inflammation in the nasal mucosa, are associated with greater sensitivity to irritants such as carbon dioxide and acetic acid (Shusterman and Murphy 2007; Shusterman et al. 2003). In the former case, the compromised function of this sensory system may represent an even more significant deficit than that of the olfactory system, as the trigeminal system is responsible for our ability to detect irritants and initiate reflexes that protect lung function.

The profound and complex nature of the airborne exposures at the WTC site, coupled with early reports of inflammation in the upper respiratory tract and the nasal passages in exposed individuals, led us to hypothesize that olfactory acuity would be decreased but that trigeminal sensitivity might be increased or decreased in this group. The goal of this study was to evaluate chemosensory function in a group of WTC-exposed individuals and compare their performance with an age-, sex-, and job title-matched comparison group having had no exposure to the WTC site. We also sought to document inflammatory or congestive factors that might have been associated with loss of sensory function.

## Participants

Beginning in September 2003, individuals who were evaluated at the Mount Sinai Hospital location in New York as part of the WTC Worker and Volunteer Medical Screening Program were provided with information about an additional chemosensory function study and given a number to call if they were interested in participating. In addition, many of the individuals evaluated earlier who had indicated they were willing to participate in additional research studies were recontacted. Between these two sources of potential participants, 102 (9 females and 93 males; mean age 45.8 ± 9.1 years) agreed to return to be tested. Testing took place on 1 or 2 days per week over a 1.5-year period. To control for the confounding effects of occupation and age on chemosensory function, a comparison group of 94 individuals (9 females and 85 males; mean age 42.2 ± 9.9 years) was also recruited and tested at the Monell Center in Philadelphia, Pennsylvania. This group was matched on a case-by-case basis for exact job title, age, sex, and smoking status, but had no exposure to the WTC site. (See Table 1 for occupational breakdown with specific job titles to which we matched participants to controls.) All individuals had been working in that job title for a minimum of 5 years; differences in years worked in that job title between matched pairs did not exceed 3.5 years. WTC workers with a history of head injury (*n* = 2) or chronic allergies (*n* = 8) were excluded from the study because of the difficulty in matching these individuals with appropriate controls. None of the individuals had been tested for chemosensory function previously and only two individuals in the WTC cohort and three in the control group reported having a sense of smell that was “a little worse than most people” during the screening.

All participants consented to the study, using a form approved by the University of Pennsylvania Institutional Review Board and the Mount Sinai Institutional Review Board. Consenting and testing of non-English-speakers were conducted in the native language of the subject. Participants were compensated for their time and travel to the testing site.

## Methods

Each volunteer was tested in a single session lasting approximately 1–2 hr. The sensory evaluation consisted of three parts, based on a validated protocol for evaluating chemosensory dysfunction (Cowart et al. 1997): odor detection threshold for phenyl ethyl alcohol, a 20-item odor identification test, and irritation detection threshold (lateralization) for n-butanol. These protocols have been used extensively to evaluate chemosensory function (Dalton et al. 2003). After administration of the sensory tests, nasal inflammation and function was evaluated by measurements of nasal airway volume, mucociliary clearance, and inflammatory mediators present in nasal lavage fluid (NLF).

### Odor detection thresholds

To measure olfactory sensitivity, phenylethyl alcohol (PEA) (Sigma-Aldrich, St. Louis, MO) was diluted into an odorless diluent [polyethylene glycol 200 (PEG 200); Sigma-Aldrich] and placed into glass bottles in a 20-step semi-log dilution series, starting with a concentration of 100% vol/vol liquid. Each bottle contained 10 mL of a stimulus (or the diluent alone). When volunteers inhaled from the nosepieces affixed to each bottle, they sampled from the ~ 250-mL headspace inside each of the two bottles (one sniffing port per nostril). We determined analytical measurements using headspace gas chromatography, and obtained thresholds using a two-alternative, forced-choice, two-up and one-down staircase method with a five-reversal criterion, as has been described previously (Dalton et al. 2000). On each trial, subjects were presented with two sets of bottles in random order: One set contained the odorant stimulus and the other contained only the diluent (blanks). After sniffing from each set sequentially, subjects were asked to identify the bottle containing an odor. No recognition or quality identification was required. An incorrect detection on any trial resulted in the presentation of the next-higher concentration, whereas two consecutive correct detections resulted in the presentation of the next-lower concentration. Five reversals were required for threshold determination, and thresholds were calculated as the geometric mean of the last four reversal concentrations. Testing was terminated under four different conditions: *a*) five reversals were achieved, resulting in a threshold value threshold; *b*) a subject failed to correctly detect the stimulus at the highest concentration; *c*) the subject correctly identified the stimulus on four consecutive trials at the lowest concentration; or *d*) the subject did not meet the criteria for threshold determination before the 40th trial (which occurred only once for a WTC worker on the odor threshold test).

### Irritation detection (lateralization) thresholds

In addition to the ability to detect odor, another significant functional capacity of the nose is to detect irritation or pungency from chemicals that stimulate the free nerve endings of the trigeminal nerve. The lateralization method for measuring irritation is a psychophysical technique that permits an objective assessment of intranasal irritation that is not confounded by negative responses to the perception of an odor (Doty et al. 2004). The lateralization method relies on the fact that chemical irritation (e.g., pain, tingling, burning, stinging, or prickling) is mediated by the somatosensory system and thus produces sensations that can be localized to one or the other nostril, whereas pure odor stimuli cannot be localized. On every trial, the individual sniffs simultaneously from two bottles—one bottle containing n-butanol dissolved in a diluent and the other diluent alone—and judges which side was exposed to the chemical. The nasal irritation threshold is therefore operationally defined as the concentration at which individuals are reliably able to judge which side of their nose is exposed (Kobal et al. 1989; Wysocki et al. 1997). To determine the irritation threshold, n-butanol (Sigma-Aldrich) was diluted into PEG 200 and placed into glass bottles in an 18 tertiary-step dilution. Thresholds were determined using the same two-up, one-down staircase method with a five-reversal criterion used to obtain olfactory thresholds.

### Odor identification

Odor identification is the ability to produce or select the correct name/label of an odorant when it is presented at a concentration well above the detection threshold. The odor identification assessment consisted of 20 common odors, each presented twice [for details, see Dalton et al. (2003)].

Each odor presentation was associated with a choice of four pictures accompanied by word identification labels. After the presentation of each odor, the subject was shown the appropriate answer card and asked to identify which of the four choices on the card best corresponded to the presented odor. Because of the inherent difficulty in the identification task, subjects were permitted to take more than one sniff before choosing an answer.

### Assessment of nasal inflammation

#### Rhinometry

Nasal patency was evaluated using an acoustic rhinometer (Eccovision; Hood Laboratories, Pembroke, MA), to determine the nasal volume as well as the cross-sectional area for the left and right nasal cavity of each participant, using methods for calibration and measurement previously described (Opiekun et al. 2003).

#### Nasal lavage

NLF samples from each subject were collected from one nostril. Subjects were given a sterilized, metered-pump aerosolizer filled with 0.1 M sterile phosphate-buffered solution without calcium or magnesium. Each pump action delivered 100 μL of solution. Subjects were asked to spray and sniff four or five times into one nostril while occluding the other nostril and then to forcibly expel the nasal contents into a glass container. Collected NLF was then centrifuged at 9,000 rpm for 10 min and the supernatant was frozen at −20°C until used for cytokine determination. Freezing NLF samples precipitates the mucins. On the day of the assay, samples were thawed completely, vortexed, and centrifuged at 1,500 × *g* (3,000 rpm) for 15 min.

#### Inflammatory mediators from nasal lavage

To evaluate whether exposure to the WTC site and subsequent alterations in chemosensory function were associated with increased levels of chronic nasal inflammation, we analyzed the collected NLF for the presence of multiple inflammatory biomarkers using multiplex cytokine assays. Multiplex technology allows for the simultaneous measurement of multiple analytes in a single sample, saving time, labor, and sample volume simultaneously, using the Beadlyte Human 9 and 10-plex Multi-Cytokine Detection System (Catalog No. 48-509 and 48-510, respectively; Millipore, Billerica, MA) and read on the Luminex-100 system version 1.7 (Luminex, Austin, TX). Analyses were carried out in the Radioimmunoassay and Biomarkers Core Facilities at Diabetes and Endocrinology Research Center in the University of Pennsylvania. NLF samples were diluted 1:1 with Beadlyte Serum Diluent. The reagents were pretested and qualified by the manufacturer to ensure the absence of cross-reactivity among antibody-coated beads. The kit was run in accordance with the manufacturer’s instructions and using the provided standards. Raw data (mean fluorescent intensity) from all kits were analyzed by Luminex beadlyte analysis software to obtain concentration values. A five-parameter regression formula was used to calculate the sample concentrations (picograms per milliliter) from the standard curves.

#### Mucociliary transport

Mucus clearance through the nasal cavity is both a function of mucus rheology and ciliary beat frequency and transport time and can be altered by numerous disease states or after exposure to airborne pollutants (Proctor et al. 2001). Mucociliary transport was evaluated using the saccharin transit time test (Anderson and Lundqvist 1977; Lale et al. 1998), in which a 1.0-mm particle of saccharin is placed via direct visual examination onto the septal surface of the inferior turbinate and the time until the volunteer reported tasting saccharin is monitored. In this study, the saccharin particle was always placed in the nostril that was not sampled for NLF, as irrigation of the nasal mucosal surface that occurs during nasal lavage could alter saccharin transit time.

### Exposure assessment

The exposure history and health screening questionnaire administered as part of the Mount Sinai Worker and Volunteer Medical Screening Program enabled us to relate the chemosensory measures to exposure and health history variables relevant to chemosensory dysfunction. Individual exposures were based on responses to questions selected *a priori* from this questionnaire. Variables of potential interest included job title, location and dates worked, duration of work per day, and respirator use, as summarized in Table 2. Of particular interest was whether the individual was in lower Manhattan on 9/11 and whether the subject was exposed to the dust cloud from the collapse of the towers, because this exposure has been associated with new-onset respiratory problems (Brackbill et al. 2009). Approximately 44% of the WTC group reported being in lower Manhattan on 9/11, and 97% of the WTC group worked on the site between 12 September and 18 September.

### Statistical analyses

Psychophysical test results for both groups were analyzed by comparing the test results for odor detection, irritation detection, and odor identification with normative data (Cowart et al. 1997) to determine the percentage of individuals whose scores fell below the normal range of function (Table 3). For congestive status and mucociliary transit, the values obtained were referenced against published values (Liote et al. 1989). We calculated prevalence ratios and 95% confidence intervals (CIs) using log-binomial regression analysis for all degrees of olfactory loss, irritant sensitivity loss, and impairment of odor identification ability. For comparisons of the concentration of inflammatory mediators in the nasal lavage samples between the WTC workers and the control group, independent *t*-tests (Statistica 8.0; StatSoft, Inc., Tulsa, OK) were performed using the Holm–Bonferroni correction for multiple comparisons.

Multivariate logistic regression (Statistica 8.0) was used to determine the association between chemosensory function classification and exposure variables such as dates and duration of work at the WTC site, job titles, areas worked, and exposure to fumes, dusts, and smoke from fires. Additional factors we explored were the use of respirators, age, and smoking status. All tests were evaluated for significance at the *p* = 0.05 level unless otherwise indicated.

## Results

### Sensory function: WTC workers versus matched controls

For each chemosensory and nasal function test, Table 3 depicts the ranges of normal function and the percentage of individuals in each group whose scores or values fell below the normal range. Odor threshold testing found significantly more individuals in the WTC cohort whose odor detection thresholds were below the normal range (i.e., below dilution step 7 for PEA) than among the comparison group, with a prevalence ratio of olfactory decrement associated with exposure at the WTC site of 1.96 (95% CI, 1.2–3.3). The decrement for the irritation threshold was more pronounced, with nearly 75% of the WTC exposed group exhibiting scores below the normal range (i.e., below dilution step 4 for n-butanol) compared with 23% in the control group. The prevalence ratio of decreased nasal irritant sensitivity associated with exposure at the WTC site was 3.28 (95% CI, 2.7–3.9).

There were no statistically significant differences between the prevalence of odor identification scores below normal range between the groups, although there were a few more individuals in the WTC group with extremely low scores than in the comparison group (8% vs. 1%, respectively). However, the relative prevalence of odor identification decrement associated with WTC exposure was nonsignificant: 1.15 (95% CI, 0.7–1.89).

We also did not observe significant differences overall in the rhinometric measures of congestive status (airway volume or minimum cross-sectional area) or mucociliary transit time between the two groups, although there were more individuals in the WTC group than among the controls who reported never tasting the saccharin particle (14 vs. 5 individuals, respectively).

### Inflammatory markers from NLF

NLF samples for both groups were analyzed in duplicate for interleukin (IL)-1α, IL-1β, IL-2, IL-4, IL-6, IL-8, IL-13, IL-15, human interferon-inducible protein 10 (IP-10), IL-12 p40, epidermal growth factor (EGF), growth-regulated oncogene (GRO), granulocyte colony-stimulating factor, macrophage inflammatory protein-1α, eotaxin, granulocyte macrophage colony-stimulating factor, monocyte chemotactic protein-1, RANTES, and tumor necrosis factor-α. The markers having more than 10% of the samples above the limit of detection, including IL-1α, IL-6, IL-8, EGF, RANTES, MCP-1, and IP-10, were analyzed further, and the results are depicted in Table 4. Between the workers and comparison groups, there were significant differences in concentration for five of the eight mediators. For example, concentrations of IL-8 were significantly higher in the WTC group than in the unexposed comparison group (765 vs. 342 pg/mL; *p* = 0.00003), as were concentrations of IL-1α, RANTES, and EGF. In contrast, concentrations of IL-6 were lower in the WTC group than in the comparison group.

### Impact of type and duration of WTC exposure on chemosensory dysfunction

Among the WTC worker cohort, we found no evidence of an association between an individual’s long-term work history at the WTC site, their prior health history, and any of their test results: exposure duration, location worked, job title, and history of respirator use were not associated with any of the sensory or functional measures. Moreover, although age was predictive of odor sensitivity among the controls, age was not associated with sensitivity among the WTC-exposed cohort. Smoking status was not predictive of sensory function in either group; however, only seven individuals in each cohort were current or past (within the preceding 10 years) smokers.

**Figure 1:**
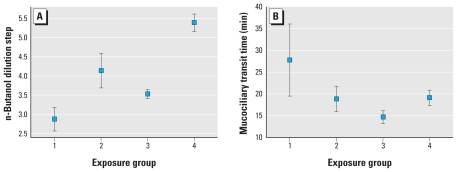
Relationship between exposure to dust cloud on 9/11 and (*A*) mean threshold (± SE) for detection of nasal irritation from n-butanol, and (*B*) mean (± SE) mucociliary transit time. Group 1: person was directly in the dust cloud from the collapse of the towers (*n* = 22); group 2: person was exposed to some/significant dust, but not in the cloud (*n* = 22); group 3: person was not exposed to dust (all but one was not present in lower Manhattan on 9/11 (*n* = 57); group 4: matched controls with no WTC exposure.

Because dust cloud exposure on 9/11 was found to be significantly associated with new-onset asthma (Brackbill et al. 2009), we were interested to learn whether this variable was associated with any chemosensory measures. We therefore grouped the WTC cohort according to their self-reports of exposure on 9/11, yielding three groups: those who were directly in the dust cloud of the collapse of the towers (*n* = 22), those who were in lower Manhattan on 9/11 with some or significant dust exposure, but not in the cloud (*n* = 22), and those who were not in lower Manhattan on 9/11 (*n* = 57). For comparison, we also included the matched controls. This classification was significantly associated with the ability to detect irritation from butanol [*F*_(3,175)_ = 13.9; *p* < 0.001] and nearly significantly associated with mucociliary transit time (*p* = 0.07). As shown in Figure 1A, the ability to detect the nasal irritancy was lowest for the 22 individuals who were directly in the dust cloud of the collapse of the towers. Thresholds for workers who experienced some dust on 9/11 did not differ from those who were not in lower Manhattan on 9/11, but those two groups were significantly lower than the matched controls. Similarly, mucociliary transit time was longest for those individuals who were directly exposed in the dust cloud (Figure 1B).

## Discussion

Although often overlooked as a locus for toxic exposure effects, the nose represents one of the first sites of impact for airborne pollutants. As such, it not only serves to trap and detoxify numerous forms of potential toxicants but performs many sensory functions equally critical to human health and safety. The impact of exposure to volatile chemicals, particulates, and metal fumes on olfaction can take many forms, ranging from total loss (anosmia) to diminished sensitivity (hyposmia) or distortions in quality (dysosmia). The mechanisms underlying the adverse effects after acute or chronic exposure may also differ, depending on the agent or agents involved in the exposure. Some agents are capable of directly damaging tissue in areas that contain high densities of olfactory receptors. Other nonreactive agents can impair olfaction indirectly through stimulation of inflammatory mediators that possibly interfere with normal signal transduction in the olfactory epithelium or that produce anatomical changes altering the conduction of the chemical molecules to the olfactory epithelium (Dalton and Opiekun 2006). In the case of the WTC workers, however, further analysis of nasal tissue is required to better identify the exact mechanism underlying the loss of function. In addition, identification of the agent(s) responsible for the damage can only be inferred from air samples collected during this period and retrospective self-reports on their work history.

Although we matched the controls to the WTC workers on a case-by-case basis for exact job title, age, sex, and smoking status, the controls were located in the Philadelphia area, whereas most of the WTC workers came from the New York City region. We did not believe this biased the results, however, because air quality data for typical pollutants collected in both regions strongly confirm the similarity of the ambient exposures, with the Philadelphia region having slightly higher levels of volatile organic compounds (Kleinman et al. 2002).

Despite observing substantial impairments in olfactory and trigeminal sensitivity among the WTC workers and volunteers relative to the occupationally matched controls, we did not find a significant difference in odor identification ability between the groups. In prior studies evaluating the effects of chemical exposure on olfactory ability, odor identification performance appeared to be inconsistently sensitive to pollutant exposure. For example, Calderon-Garciduenas et al. (2010) found decrements in odor identification ability among children and young adults living in Mexico City. However, in two studies comparing residents of Mexico City with residents from an unpolluted area, Hudson et al. found significant differences in threshold sensitivity (Guarneros et al. 2009; Hudson et al. 2006), but no effects on odor identification (Guarneros et al. 2009). Schwartz et al. (1989) found cumulative exposure effects on odor identification among acrylate-exposed workers only when analyzed using a nested case–control design; overall, there were no significant differences among the exposed workers and matched controls. However, we also note that in our study, between 17% and 20% of individuals in each group performed well below normal on the odor identification test. This prevalence is greater than what would be expected in the general population and may reflect exposure-induced damage related to their occupations, as nearly half of our sample were employed in the construction trades. The additional exposure at the WTC site may not have contributed substantially to odor identification deficits beyond that. Still, there were eight individuals in the WTC group whose odor identification ability was severely impaired and who were also classified as anosmic on the odor threshold test, a higher frequency than observed among the matched controls.

Differences in the levels of cytokines/chemokines in the NLF suggest that inflammatory mechanisms may underlie the functional effects we observed. As IL-6 is one of the most important mediators of the acute phase of response to injury, the lower levels found among the WTC workers with chronic inflammatory symptoms would not be considered unusual (Wang et al. 1997). Increased levels of IL-8, on the other hand, are frequently found in cases of chronic inflammation of the nasal cavity, which is also consistent with the data (Barraza-Villarreal et al. 2008; Raulf-Heimsoth et al. 2007).

The robust association we found between the degree of exposure to the dust cloud on 9/11 and the loss of trigeminal sensitivity is particularly noteworthy, given that individuals were tested > 2 years after this exposure. Individuals who were not directly exposed to the dust cloud (either because of distance from the towers or by not being in lower Manhattan on 9/11) had significantly decreased ability to detect irritation relative to the matched controls. However, the thresholds of those individuals who were directly exposed in the dust cloud showed the most dramatic signs of impairment. The relationship between trigeminal function and pollutant exposure has not been evaluated as often as that for olfactory function, but should be considered in future studies.

The failure to find a predictive relationship between long-term exposure history at the WTC site and performance on chemosensory function tests was surprising. However, we note that 97% of our test population worked/volunteered in lower Manhattan between 12 September and 18 September, which, after 9/11, was the period during which the potential for pollutant exposure was likely at its highest. If the chemosensory alterations we observed were due primarily to dust exposure on 9/11 and to some degree to other exposures during the first week, then the lack of variance in our exposure data during this week would obscure finding a relationship. We have noted that among the matched controls, sensitivity to the odor of PEA and the irritancy of n-butanol was associated with age. No such relationships were observed among the WTC workers, as the relatively small impact of this variable may have been overshadowed by damage incurred from early exposure at the site.

Although the nasal chemosensory system can be remarkably robust to damage from airborne pollutants (Ruitenberg and Vukovic 2008), its well-documented regenerative powers can be overwhelmed by certain types of acute or chronic exposures. We undertook this study to determine whether the exposures experienced by individuals in lower Manhattan on 9/11 and those who worked and volunteered in the days, weeks, and months afterward produced long-lasting chemosensory deficits from exposure to the volatile gases, fumes, and dusts associated with that site. Although many of the WTC workers and their matched controls were engaged in occupations where some level of chemosensory dysfunction could be expected to occur, the significantly higher prevalence of chemosensory dysfunction in the WTC group leads us to conclude that the profound exposures experienced in lower Manhattan increased the risk of dysfunction beyond that associated with the workers’ regular occupations.

Notably, the near-absent ability to detect nasal irritation for n-butanol for those individuals who were caught in the dust cloud from the collapse of the towers may be the most significant hazard we documented, as it is this sensory system that is in the first line of defense against many toxicants that could otherwise reach and potentially damage the lower airways. However, the decreased sensitivity to the irritancy of n-butanol may not be predictive of deficits for detecting all nasal irritants. Since the cloning of the capsaicin receptor in 1997, researchers have identified a number of transient receptor potential ion channels on sensory neurons, which appear to confer some level of specificity in the neuronal response to airway irritants (Bessac and Jordt 2008). Nonetheless, the profoundly decreased sensitivity to n-butanol was not due to specific exposure to this compound, so the deficiency likely represents a more widespread decrement in trigeminal sensory response. Future evaluations should include tests using different classes of airway irritants to evaluate whether exposed individuals sustain a general loss of trigeminal sensitivity.

It is unknown to what degree, if any, recovery of olfactory or trigeminal function has occurred in the interim for these individuals. Nor is it known how many of the WTC-exposed individuals who were not tested in this study have suffered similar chemosensory impairment but who may be unaware that they have lost an important sensory tool for detecting airborne hazards. For this reason, broader screening of chemosensory function among WTC-exposed individuals seems warranted, with a special focus on reevaluating individuals who participated in this study. The goal of such efforts should be not only to determine the prevalence or degree of chronic impairment for these individuals, but also to inform us of the potential for recovery, the factors that may be associated with recovery, and the time course necessary for recovery of function. Moreover, the lessons learned from studying this cohort can also provide important clues for monitoring and protecting the upper airways and chemosensory function in other populations having potential for acute exposure to airborne toxicants.

## Figures and Tables

**Table 1:** Participant job title categories (*n*).

Occupation	WTC	Control
Office/Indoor	24	17

Attorney	1	1
Banker	1	1
Chef	1	1
Correction officer	2	2
Customer service	3	1
Network designer	1	1
Salesperson	4	2
Security	2	2
Teacher	1	1
Social worker	1	1
Telecom technician	2	1
NYC transit clerk	5	3

Construction/maintenance	48	48

Asbestos remover	7	7
Carpenter	5	5
Diesel mechanic	3	3
Electrician	3	3
Engineer	1	1
Iron worker	11	11
Road repair	8	8
Building maintenance	5	5
Sanitation worker	3	3
Truck driver	2	2

EMS/responder	30	29

Firefighter	2	2
Detective	6	6
Police	16	15
EMS responder	5	5
Red Cross worker	1	1

**Table 2:** Summary of selected self-reported exposure history at the WTC site, including the percentage of participants in the cohort contributing to each variable.

Exposure history	Percentage of participants	Mean ± SD
Hours worked 11 September 2001	44	10.5 ± 4.5
Days worked 11 September–18 September 2001	97	4.6 ± 2.4
Days worked October 2001	59	22.3 ± 13.7
Days worked November/December 2001	50	35.4 ± 17.5
Days worked January–June 2002	39	69.3 ± 44.7
Wore respirator at any time	56	

**Table 3:** Results and value ranges for normosmic/healthy nasal function.

Test	Purpose	Normal range	Results		Prevalence ratio^a^ (95% CI)
			WTC	Controls	
Odor threshold (PEA)	Olfactory nerve (cranial nerve 1) function	Dilution step ≥ 7	22% < normal 9% anosmic 13% hyposmic	10% < normal 3% anosmic 7% hyposmic	1.96 (1.2–3.3)
Irritant threshold (n-butanol)	Trigeminal nerve (cranial nerve 5) function	Dilution step ≥ 4	74% < 4	23% < 4	3.28 (2.7–3.9)
Odor identification	Cranial nerve 1 and central olfactory function	Males ≥ 31/40 Females ≥ 34/40	20% < normal 8% severe	17% < normal 1% severe	1.15 (0.7–1.89)
Mucociliary transit test	Impaired nasal clearance from damaged cilia or mucus rheology	≤ 30 min	19% > 30 min	20% > 30 min	0.95 (0.6–1.78)
Acoustic rhinometry	Nasal congestion or obstruction	Mean cross-section area: 0.35–0.65 cm	25% < 0.35	21% < 0.35	1.07 (0.5–1.94)

a Each ratio refers to the prevalence of all individuals scoring below normal for each outcome.

**Table 4:** Concentrations of cytokines/chemokines from NLF.

Marker	WTC workers Mean (pg/mL) ± SE	Controls Mean (pg/mL) ± SE	*p*-Value
EFG	200.1 ± 10.1	160.1 ± 11.3	0.009*
IL-1α	74.6 ± 5.3	51.3 ± 4.1	0.0009*
IL-6	20.9 ± 2.9	54.6 ± 7.9	0.00002*
IL-8	765.9 ± 86.1	342.8 ± 35.2	0.00003*
MCP-1	29.2 ± 2.4	35.8 ± 3.8	0.13
RANTES	67.6 ± 23.2	14.9 ± 2.2	0.044
GRO	1843.7 ± 171.5	3167.7 ± 289	0.00005*
IP-10	1896.7 ± 90.2	1082.3 ± 91.5	0.15

* Significant at the Holm–Bonferroni corrected *p*-value.
